# The Relationship Between Relative Value Units and Outcomes: A Multivariate Analysis of Plastic Surgery Procedures

**Published:** 2012-12-27

**Authors:** Khang T. Nguyen, Michael S. Gart, John T. Smetona, Apas Aggarwal, Karl Y. Bilimoria, John Y. S. Kim

**Affiliations:** ^a^Northwestern University Feinberg School of Medicine; ^b^Division of Plastic and Reconstructive Surgery, Department of Surgery, Northwestern Memorial Hospital; ^c^Division of Surgical Oncology, Department of Surgery, Northwestern Memorial Hospital, Chicago, Ill

## Abstract

**Introduction:** Relative value units (RVUs) were developed as a quantifier of requisite training, knowledge, and technical expertise for performing various procedures. In select procedures, increasing RVUs have been shown to substitute well for increasing surgical complexity and have been linked to greater risk of complications. The relationship of RVU to outcomes has yet to be examined in the plastic surgery population. **Methods:** This study analyzed nearly 15,000 patients from a standardized, multicenter database to better define the link between RVUs and outcomes in this surgical population. The American College of Surgeons’ National Surgical Quality Improvement Program was retrospectively reviewed from 2006 to 2010. **Results:** A total of 14,936 patients undergoing primary procedures of plastic surgery were identified. Independent risk factors for complications were analyzed using multivariable logistic regression. A unit increase in RVUs was associated with a 1.7% increase in the odds of overall complications and 1.0% increase in the odds of surgical site complications but did not predict mortality or reoperation. A unit increase in RVUs was also associated with a prolongation of operative time by 0.41 minutes, but RVUs only accounted for 15.6% of variability in operative times. **Conclusions:** In the plastic surgery population, increasing RVUs correlates with increased risks of overall complications and surgical site complications. While increasing RVUs may independently prolong operative times, they only accounted for 15.6% of observed variance, indicating that other factors are clearly involved. These findings must be weighed against the benefits of performing more complex surgeries, including time and cost savings, and considered in each patient's risk-benefit analysis.

The relative value unit (RVU) was developed as a quantifier of physician labor, technical skill, medical decision making, and training time required to complete medical and surgical procedures.^1^^-^4 The RVU has therefore become a commonly used metric of surgical complexity,^5^ and the relationship of RVUs to complications has been studied in pediatric, vascular, and hepatobiliary surgery.^6^^-^8 While it is generally assumed that complication rates increase in parallel with surgical complexity, this relationship has not been studied in the plastic surgery population. This study aims to determine the relationship of RVUs, used as a surrogate for surgical complexity, and surgical outcomes in plastic and reconstructive surgery.

In light of ongoing US healthcare reforms, the RVU has taken on even greater significance. Medicare reimbursements for medical and surgical procedures have been based on RVUs since 1992,^9^^-^12 with higher RVUs generally corresponding to higher reimbursements. Thus, RVU may represent a paradoxical link whereby potentially poorer patient outcomes are reimbursed at a higher rate, increasing overall health care expenses when treatment of resulting complications is also considered. Recent and upcoming policy changes toward outcomes-based reimbursement may affect the valuation of some procedural services.^13^

Large studies evaluating the link between surgical RVUs and postoperative outcomes, particularly in plastic and reconstructive surgery, have been logistically challenging due to the lack of a universal, objective outcomes database that captures both data points. The American College of Surgeons’ National Surgical Quality Improvement Program (NSQIP) has filled this data void by sampling surgical RVUs, patient demographics, and outcomes data in a single, standardized database from more than 240 institutions across the United States.^5^^,^^14^^-^16 This unique database provides a sufficient sample size to evaluate the relationship between RVUs and outcomes in plastic and reconstructive surgery in a statistically powerful manner. This study utilizes the NSQIP database to develop a multivariate model of independent risk factors, including RVUs, for complications following plastic and reconstructive surgery.

## METHODS

The NSQIP database was retrospectively reviewed to obtain data on all patients undergoing a primary procedure performed by a plastic and reconstructive surgeon between 2006 and 2010. As defined by NSQIP, the primary procedure was the most complex procedure undertaken during a surgery. Surgical complexity was measured by summing RVUs for the primary procedure and all concomitant procedures performed in the same operative setting. The surgical subspecialty undertaking nonprimary procedures was not tracked by NSQIP.

Primary endpoints tracked by NSQIP include overall complications, surgical site complications, reoperation, and mortalities within a 30-day follow-up period after the original procedure(s). On the basis of NSQIP definitions, surgical site complications included the following: superficial, deep, or organ-space surgical site infection; wound disruption; graft/prosthesis/flap failure. Nonsurgical site complications included the following: transfusion of more than 4 units of blood; deep venous thrombosis, pulmonary embolism, unplanned intubation, pneumonia, mechanical ventilation for more than 48 hours, myocardial infarction, cardiac arrest, renal insufficiency, acute renal failure, urinary tract infection, cerebrovascular accident, peripheral neurologic deficit, coma lasting more than 24 hours, and sepsis/septic shock. Overall complications encompassed both surgical and nonsurgical site complications. *Mortality* was defined as death from any cause within the 30-day follow-up period, and reoperation was defined as a return to the operating room within 30 days of the original procedure. Operative duration, recorded in minutes, was tracked as a secondary endpoint.

Multivariate logistic regression was used to model risk factors associated with primary endpoints for plastic surgery procedures. Preoperative variables describing patient demographics, comorbidities, and operative variables were analyzed for association with each primary endpoint using bivariate screening. Dichotomous and continuous variables were tested using chi-squared (χ^2^) test and the Student *t* test, respectively. All variables showing an association with a primary endpoint at a significance level of *P* < .2 were entered into a backwards stepwise regression for that endpoint with exit criteria of *P* < .05. Variables associated with fewer than 10 incidences of an outcome were excluded from logistic regression models.^17^^,^^18^ In addition, variables for which data was not available for more than 5% of patients were excluded.

Variables meeting stepwise regression exit criteria for each primary endpoint were entered into multivariate logistic regression to calculate odds ratios. C-statistic values were calculated to assess discrimination of each logistic regression model.^19^^,^^20^ Linear regression was used to model the association between RVUs and operative time. All statistical analyses were performed using SPSS version 20.0 (SPSS, Inc, Chicago, Illinois).

## RESULTS

Of the approximately 1.3 million patients in the 2006-2010 NSQIP database, 14,936 had a primary plastic surgery procedure with RVU data. The mean age of patients was 48.42 ± 15.43 years. Among those, 3034 were male (20.3%); and 10,823 were Caucasian (72.5%). Summary demographic and clinical characteristics of this cohort are presented in Table 1.

The rate of overall complications was 6.59% (n = 981). Surgical site complications were noted in 4.04% of patients (n = 603). Superficial infections were the most commonly observed surgical site complication (n = 287), followed by infection of deep incisions (n = 121), graft/flap failure (n = 99), wound disruption (n = 94), and organ space infections (n = 53). The 30-day reoperation and mortality rates were 4.79% (n = 716) and 0.33% (n = 50), respectively. The mean operative time was 135.0 ± 107.1 minutes. This data is presented in Table 2.

The mean number of total RVUs for all patients was 17.91 (range: 0.30–177.64); for patients undergoing a primary procedure alone, the mean was 12.44; and for patients undergoing multiple, simultaneous procedures, the mean was 33.23. On average, the primary procedure accounted for 55% of total RVUs for patients undergoing multiple simultaneous procedures.

Patient risk factors for overall complications include the following: ASA (American Society of Anesthesiologists) class ≥3 (OR = 1.838), increasing body mass index (BMI) (OR = 1.032), chronic steroid use (OR = 1.613), metastatic cancer (OR = 2.527), nonindependent functional status (OR = 1.580), preoperative delirium (OR = 2.593), preoperative wound infection (OR = 1.587), prior surgery within 30 days (OR = 1.547), and ventilator dependency (OR = 5.017). Intraoperative risk factors for overall complications included surgical house-staff assistance (OR = 1.353), transfusion of more than 4 units of blood (OR = 2.481), and total RVUs (OR = 1.017). Ambulatory surgical procedures were associated with a decreased risk for complications (OR = 0.334). These data are presented in Table 3.

Patient risk factors for surgical site complications included the following: ASA class ≥3 (OR = 1.456), BMI (OR = 1.042), chronic steroid use (OR = 1.684), history of transient ischemic attacks (OR = 1.949), and preoperative wound infection (OR = 1.489). Intraoperative risk factors for surgical site complications included surgical house-staff assistance (OR = 1.403), and total RVUs (OR = 1.010). Ambulatory surgical procedures were associated with a decreased risk of surgical site complications (OR = 0.477). These data are presented in Table 4.

Patient risk factors for reoperation include the following: ASA class ≥3 (OR = 1.281), hypertension (OR = 1.331), nonindependent functional status (OR = 1.335), preoperative sepsis (OR = 1.823), preoperative wound infection (OR = 1.980), prior surgery within 30 days (OR = 2.017), and renal failure (OR = 3.737). A history of cardiac surgery (OR = 0.663), and non-Caucasian race (OR = 0.785) were associated with a decreased risk of reoperation. Surgical house-staff assistance (OR = 1.472) was the only intraoperative factor found to predict an increased risk for reoperation; ambulatory surgical procedures (OR = 0.352) were associated with a decreased risk for reoperation. A summary of risk factors for reoperation is presented in Table 5.

Patient risk factors for increased mortality included increasing age (OR = 1.063), nonindependent functional status (OR = 5.427), preoperative sepsis (OR = 6.255), preoperative wound infection (OR = 2.306), and previous coronary revascularization (OR = 2.484). No intraoperative variables were significantly associated with mortality. A summary of risk factors for mortality is presented in Table 6.

A single unit increase in RVU was associated with a 0.410-minute increase in operative time. Relative value units predicted 15.6% of the variability in operative time (*r*^2^ = 0.156).

## DISCUSSION

Procedural RVUs correlate highly with surgical complexity.^5^ Reports have linked increasing surgical complexity (RVUs) to respiratory failure following pediatric surgery,^6^ surgical site infections following general and vascular surgery,^7^ and complications following liver resection.^8^ To our knowledge, there have been no studies linking surgical complexity, as measured by RVUs or any other metric, to outcomes in plastic and reconstructive surgery. In this study, multivariate analysis showed that RVUs are positively associated with risk of complications following plastic and reconstructive surgery independent of patient-related and intraoperative factors.

### Patient-related risk factors for complications

Multivariate regression modeling identified a number of patient-associated variables that were significantly associated with an increased risk of complications or reoperation. Many of these factors are not surprising and in line with previously published reports, including increasing age, ASA classification, and BMI, and also chronic steroid use. Increasing age, BMI, and ASA classification are well-known risk factors for complications in various surgical specialties, including plastic and reconstructive surgery.^21^^-^24 Chronic steroid use is thought to impair wound healing and increase the risk of postoperative wound infection.^25^^-^27 Other factors associated with increased risks of complications or reoperation included poor baseline functional status, preoperative wound infections or sepsis, metastatic malignancy, history of transient ischemic attack (TIA), poor baseline functional status, previous coronary revascularization, and recent surgery. Although these variables have not been extensively studied as independent risk factors for postoperative complications,^14^^,^^28^^,^^29^ they suggest severe underlying pathologies, such as systemic illness, vascular insufficiency or advanced atherosclerosis, immunosuppression, malnutrition, autonomic instability, or other pathologic processes that may predict poorer surgical outcomes in these patients.

Interestingly, a history of previous cardiac surgery was found to predict a lower rate of reoperation. One potential explanation for this might be reluctance of surgeons to reoperate on a patient with a significant history of heart disease in a situation where a healthier patient might benefit from reoperation. For example, a wound dehiscence that might be closed by primary or delayed primary intent in a healthy patient might be left to heal secondarily in a patient with significant cardiac comorbidities to avoid the risk of additional anesthetic.

### Intraoperative risk factors for complications

As might be predicted, ambulatory surgical procedures were associated with a dramatic 66.6% reduction in the odds of overall complications and a 52.3% reduction in the odds of surgical site complications compared with inpatient procedures. This indirectly supports the idea that more complex surgeries, which are usually undertaken in an inpatient setting, may lead to poorer outcomes. However, factors such as increased risk of nosocomial infections and higher prevalence of multidrug resistant organisms in inpatient settings may confound this analysis.^29^^,^^30^

Resident participation in surgery predicted a 35.3% increase in the odds of overall complications and 40.3% increase in the odds of surgical site complications. This unexpected finding has several potential explanations. One logical explanation might be the prolongation of operative times associated with intraoperative house-staff teaching, for both surgical and anesthesiology residents. Another might be inexperience and technical error on the part of the trainee. This is certainly an area for future investigation across all surgical disciplines.

### Predictors of mortality

As noted earlier, many of the factors associated with an increased risk of mortality are those associated with poorer baseline health and functional status. The relatively small number of mortalities in this series (n = 50 [0.33%]) precluded a robust statistical analysis of positive and negative predictors for this outcome.

### Significance of RVUs as a predictor of complications

While previous studies have used RVUs as a surrogate for surgical complexity in individual procedures, it is unclear whether summation of RVUs from concurrent procedures can similarly substitute for overall surgical complexity. However, for patients with multiple procedures, the primary procedure, in this case, all plastic surgical procedures, contributed 55% of total RVUs included in this analysis. In addition, subanalysis of only patients undergoing multiple procedures indicated that surgical complexity, as measured by total RVUs, persisted as significant risk factor for complications (data not shown). Thus, it is likely that total RVUs is a valid measure of surgical complexity for patients undergoing multiple procedures; however, this cannot be definitively stated from the present results.

It stands to reason that added surgical complexity will only increase the potential for postoperative complications; however, this conclusion is not rooted in strong evidence. Previous studies have demonstrated increased complications when complexity has been added to traditional procedures but have not *quantitatively* described the magnitude of these changes. Thus, the existing literature offers only limited insight into the relationship between surgical complexity and outcomes in plastic and reconstructive surgery. For example, lipoabdominoplasty shows a higher rate of seroma formation than traditional abdominoplasty^31^; the extent of tissue excision directly corresponds to seroma rates in body contouring procedures^32^; and facelifts performed in conjunction with additional procedures have demonstrated an increased risk of deep venous thrombosis.^33^ Similarly, immediate breast reconstruction, which is performed in conjunction with mastectomy, may lead to higher complications rates than delayed reconstructions performed as a separate procedure.^34^^-^37 In addition to their limited scope, many of these existing comparisons are drawn from single-surgeon or single-institution series, incorporating a natural selection bias and reducing generalizability. The results of the current study are derived from numerous military, community, and academic institutions across the country, which decreases selection bias as a potential confounder and broadens the applicability of our results.

We found that a single unit increase in total RVUs increased the incidence of overall complications by 1.7% and the incidence of surgical site complications by 1.0% in the 30-day postoperative period. Increasing total RVUs, a surrogate for increasing surgical complexity, may lead to longer operative times and, therefore more prolonged exposure to anesthesia, with resultant increased risk of deep venous thrombosis, arrhythmia, and other related complications.^22^^,^^33^^,^^38^^,^^39^ Linear regression indicated that a single unit increase in RVUs was associated with a 0.410-minute increase in total operative time. However, variability in total RVUs only explained 15.6% of operative time variability in this study, suggesting that this association does not tell the complete story, and other factors are likely contributing to these observed trends. Given that concomitant surgical procedures can often be performed in parallel, particularly when surgical house-staff are present to assist, it is not surprising that operative times do not increase linearly with increasing RVUs.

Although RVUs were a significant risk factor for overall and surgical site complications, they did not predict reoperations or increased mortality, suggesting that, while complex surgery may result in a higher overall rate of complications, a significant proportion of these complications might be successfully managed nonoperatively and do not significantly contribute to overall mortality.

### Limitations and additional considerations

Limitations of this study include the relatively short follow-up data provided by the NSQIP database. History of radiotherapy to the involved anatomy was not tracked, despite being a well-known risk factor of various complications following reconstructive and cosmetic procedures. Although many complications, particularly those requiring reoperation, will tend to occur in the early postoperative period, several complications, including deep venous thrombosis/pulmonary embolism (DVT/PE), seroma and wound breakdown among others, may not clinically manifest within 30 days of surgery. Moreover, this initial study is limited to procedures performed primarily by plastic surgeons. In future iterations, we can also apply this methodology to multidisciplinary operative cases. Lastly, some procedures falling under the purview of plastic surgery are not covered by Medicare and, as such, are not assigned an RVU value (ie, RVU = 0), therefore the relative contribution of these procedures cannot be included in this analysis.

The authors recognize the potential implications of linking RVUs to complications in the era of outcomes-based reimbursement. It is important to note that the valuation of procedures via RVUs includes factors beyond procedural complexity, such as training time for a surgeon to reach technical proficiency and the intensity of work required to complete the procedure. Furthermore, complex procedures may incur an acceptable increase in the risk of complications to save time, provide greater patient benefits, and reduce costs.^40^ Finally, it is plausible that high RVUs actually account for the costs associated with managing complications and that these costs vary by procedure. Thus, it would be too simplistic to argue an association with complications threatens the validity of current RVU assignments.

However, it is worthwhile to note that RVU productivity correlates with surgical department margins.^41^ Moreover, RVU productivity per unit time worked can vary dramatically across procedures and surgical specialty.^42^ The finding of RVUs as an independent risk factor for complications in our analysis raises an important health policy question: How should surgical complexity be considered in the reimbursement scheme? The answer to this question hangs in the balance of many current political agendas and will likely not be decided for some time. In the interim, further investigation of the impact of surgical complexity on postoperative complication rates in all surgical disciplines will help improve quality and safety of surgical treatment.

## CONCLUSIONS

Surgical complexity and RVUs have been linked to outcomes in many specific instances in the available literature. The use of the NSQIP database allows us to evaluate, for the first time, the relationship between RVUs in plastic surgery procedures and postoperative outcomes. In addition to providing insight on independent predictors of increased complications in plastic surgery, the current study demonstrates that increasing RVUs are linked to heightened overall complications and surgical site complications in the plastic surgery population. With ongoing—and looming—changes in health policy, this important relationship between RVUs and outcomes will undoubtedly come under greater scrutiny.

## Figures and Tables

**Table 1:** Summary of patient characteristics

Patient Characteristic	n = 14,936	%
Age	48.42 ± 15.43	—
Male	3034	20.3
Race		
White	10823	72.5
Nonwhite	4113	27.5
Body mass index	28.46 ± 7.16	—
Clinical characteristics		
Chemotherapy < 30 d	228	1.5
Prior operation within 30 d	663	4.4
Radiotherapy < 90 d	44	0.3
Smokers	2726	18.3
Steroid use	257	1.7
Comorbidities		
Bleeding disorders	275	1.8
Chronic obstructive pulmonary disease	278	1.9
Congestive heart failure	39	0.3
Diabetes	1253	8.4
Hypertension	4120	27.6
History of cardiac surgery	376	2.5
History of percutaneous cardiac intervention	314	2.1
Peripheral vascular disease	166	1.1

**Table 2:** Summary of postoperative outcomes

Outcome	n = 14,936	%
Overall complications	981	6.59
Surgical site complications	603	4.04
Surgical site infections		
Superficial SSI	287	1.92
Deep incisional SSI	121	0.81
Organ/Space SSI	53	0.35
Graft/Flap failure	99	0.66
Wound disruption	94	0.48
Reoperations within 30 d	716	4.79
Mortalities within 30 d	50	0.33
Operative time	135.0 ± 107.1	—

**Table 3:** Regression analysis of risk factors for overall complications

Risk Factor	Odds Ratio (95% CI)	*P*
Patient characteristics/Comorbidities		
ASA class ≥ 3	1.838(1.561–2.164)	<.001
Body mass index	1.032(1.023–1.040)	<.001
Chronic steroid use	1.613(1.119–2.326)	.009
Metastatic cancer	2.527(1.388–4.599)	.002
Nonindependent functional status	1.580(1.234–2.024)	<.001
Preoperative delirium	2.593(1.135–5.924)	.021
Preoperative wound infection	1.587(1.302–1.933)	<.001
Prior surgery within 30 d	1.547(1.217–1.965)	.001
Ventilator dependency	5.017(2.262–11.128)	<.001
Intraoperative factors		
Ambulatory surgery (vs inpatient)	0.334(0.284–0.393)	<.001
Surgical house-staff assistance	1.353(1.172–1.562)	<.001
Transfusion of >4 units of blood	2.481(1.228–5.015)	.023
Total RVUs/Surgical complexity	1.017(1.013–1.021)	<.001

^*^C statistic = 0.787.

**Table 4:** Regression analysis of risk factors for surgical site complications

Risk Factor	Odds Ratio (95% CI)	*P*
Patient characteristics/Comorbidities		
ASA class ≥ 3	1.456(1.198–1.771)	<.001
Body mass index	1.042(1.032–1.051)	<.001
Chronic steroid use	1.684(1.076–2.636)	.023
History of transient ischemic attacks	1.949(1.146–3.314)	.014
Preoperative wound infection	1.489(1.180–1.881)	.001
Intraoperative factors		
Ambulatory surgery (vs inpatient)	0.477(0.394–0.577)	<.001
Surgical house-staff assistance	1.403(1.195–1.666)	<.001
Total RVUs/Surgical complexity	1.010(1.005–1.015)	<.001

^*^C statistic = 0.718.

**Table 5:** Regression analysis of risk factors for reoperation

Risk Factor	Odds Ratio (95% CI)	*P*
Patient characteristics/Comorbidities		
ASA class ≥ 3	1.281(1.137–1.381)	.001
Hypertension	1.331(1.116–1.587)	.001
History of previous cardiac surgery	0.663(0.452–0.971)	.035
Nonindependent functional status	1.335(1.028–1.734)	.030
Non-Caucasian race	0.785(0.650–0.948)	.012
Preoperative sepsis	1.823(1.338–2.483)	<.001
Preoperative wound infection	1.980(1.603–2.447)	<.001
Prior surgery within 30 d	2.017(1.581–2.574)	<.001
Renal failure	3.737(1.723–8.102)	.001
Intraoperative factors		
Ambulatory surgery (vs inpatient)	0.352(0.293–0.422)	<.001
Surgical house-staff assistance	1.472(1.253–1.730)	<.001

^*^C statistic = 0.775.

**Table 6:** Regression analysis of risk factors for mortality

Risk Factor	Odds Ratio (95% CI)	*P*
Patient characteristics/Comorbidities age		
Age	1.063(1.040–1.088)	<.001
History of percutaneous cardiac intervention	2.484(1.107–5.576)	.027
Nonindependent functional status	5.427(2.449–12.028)	<.001
Preoperative sepsis	6.255(3.055–12.806)	<.001
Preoperative wound infection	2.306(1.053–5.052)	.037

^*^C statistic = 0.922.
